# Hyperspectral signature-band extraction and learning: an example of sugar content prediction of *Syzygium samarangense*

**DOI:** 10.1038/s41598-023-41603-6

**Published:** 2023-09-12

**Authors:** Yung-Jhe Yan, Weng-Keong Wong, Chih-Jung Chen, Chi-Cho Huang, Jen‑Tzung Chien, Mang Ou-Yang

**Affiliations:** 1 Institute of Electrical and Control Engineering, National Yang Ming Chiao Tung University, Hsinchu, Taiwan; 2 Institute of Biomedical Engineering, National Yang Ming Chiao Tung University, Hsinchu, Taiwan; 3 Fengshan Tropical Horticultural Experiment Branch, Kaohsiung, Taiwan

**Keywords:** Optical imaging, Optical spectroscopy, Plant sciences, Engineering, Mathematics and computing, Computational biology and bioinformatics

## Abstract

This study proposes a method to extract the signature bands from the deep learning models of multispectral data converted from the hyperspectral data. The signature bands with two deep-learning models were further used to predict the sugar content of the *Syzygium samarangense*. Firstly, the hyperspectral data with the bandwidths lower than 2.5 nm were converted to the spectral data with multiple bandwidths higher than 2.5 nm to simulate the multispectral data. The convolution neural network (CNN) and the feedforward neural network (FNN) used these spectral data to predict the sugar content of the *Syzygium samarangense* and obtained the lowest mean absolute error (MAE) of 0.400° Brix and 0.408° Brix, respectively. Secondly, the absolute mean of the integrated gradient method was used to extract multiple signature bands from the CNN and FNN models for sugariness prediction. A total of thirty sets of six signature bands were selected from the CNN and FNN models, which were trained by using the spectral data with five bandwidths in the visible (VIS), visible to near-infrared (VISNIR), and visible to short-waved infrared (VISWIR) wavelengths ranging from 400 to 700 nm, 400 to 1000 nm, and 400 to 1700 nm. Lastly, these signature-band data were used to train the CNN and FNN models for sugar content prediction. The FNN model using VISWIR signature bands with a bandwidth of ± 12.5 nm had a minimum MAE of 0.390°Brix compared to the others. The CNN model using VISWIR signature bands with a bandwidth of ± 10 nm had the lowest MAE of 0.549° Brix compared to the other CNN models. The MAEs of the models with only six spectral bands were even better than those with tens or hundreds of spectral bands. These results reveal that six signature bands have the potential to be used in a small and compact multispectral device to predict the sugar content of the *Syzygium samarangense*.

## Introduction

Taiwan is suitable for the growth of various fruit trees because Taiwan is located in the subtropical region and has diverse terrain. Fruits are produced in Taiwan all year round, and more than 30 kinds of fruits are produced in Taiwan. In 2021, The production value of the agricultural products in Taiwan was US$ 9 billion^1^. The production value of fruits was US$ 3.35 billion, ranked first among agricultural products, and accounted for 37.2% of the total agricultural products. In 2020, the top four fruit export volumes in Taiwan were pineapple, sugar apple, mango, and wax apple^2^. The wax apple, with the scientific name of *Syzygium samarangens*, has the highest export unit price of approximately US$ 3.8 to US$ 5.8. The popular species of wax apple in Taiwan include Pink^3^, Palm, Ruby, Tainung No.1 Amethyst, Tainung No.2 Big Shape, and Tainung No.3 Sugar Barbie with the highest unit price^4^.

The price of fruits depends on their quality which is related to fruit shape, size, appearance, water content, pulp texture, soluble solids, acidity, sugar content, post-harvest fresh-keeping packaging, etc. Among them, the sugar content is an essential indicator for tasting fruits and is normally measured using a refractometer. The refractometer measuring the sugar content of fruit juices is a destructive way. Thus, it can only measure the sugar content of a sample of the fruit. However, different fruits may contain different sugar content. These differences come from different trees or positions on the tree, climate conditions, and different cultivation methods. Measuring the sugar content of each fruit non-invasive and destructively would bring at least two benefits. Cultivation methods could be optimized by observing the sugar content distribution of the fruits in each tree or a single tree on the farm without destroying these fruits. The quality of the fruits can be further graded by non-destructively measuring the sugar content to increase the economic value of fruits. Thus, the better-quality fruits can have a higher price, while the lower-quality fruits can be processed as canned or preserved food.

Hyperspectral imaging (HSI) systems collecting both reflectance spectrum and image data of a sample can be developed as a non-destructive measurement^5–7^. The deterministic methods for hyperspectral analysis include the Multiple Linear Regression (MLR), Principal Component Regression (PCR), and Partial Least Square Regression (PLSR). Peris et al*.*^8^ applied the MLR with near-infrared spectral data to measure the solids content of peaches. Mendoza et al*.*^9^ used the PLSR on hyperspectral scattering data to detect apple fruit firmness. Liang et al*.*^10^ detected the zebra chip disease in potatoes using the PLSR with near-infrared (NIR) spectroscopy. Kemps et al*.*^11^ assessed the concentration of anthocyanins, polyphenols, and the sugar content in grapes by PLSR. Chuang et al*.*^12^ proposed the Independent Component Analysis (ICA) and PLSR with NIR spectral data to quantify the sugar content of wax apples. Viegas et al*.*^13^ suggested that the total anthocyanin content (TAC) and the total phenolic compounds (TPC) could be determined by the PLR with NIR spectral data. In addition to the deterministic methods, deep learning methods have been developed to predict the quality of fruits. The convolution neural network (CNN) and the support vector machine (SVM) were proposed to detect the ripeness of strawberries^14^ and the maturity of citrus^15^. Furthermore, Tu et al*.*^16^ used the region-based CNN-based model to identify and detect the number of passion fruit in orchards. Fajardo and Whelan^17^ detected the fruit in orchards using CNN-based models. Marani et al*.*^18^ obtained high accuracy of grape bunch segmentation with a deep neural network.

HSI and multispectral imaging (MSI) systems act as the important instruments to conduct non-destructively analysis and detection. HSI systems measure hundreds to thousands of spectral bands with submicron-level resolution but have the highest measurement duration in all spectral systems. Contrarily, multispectral imaging (MSI) systems detect fewer bands but with a shorter measurement duration and smaller volume compared to those of HSI systems. Thus, the HSI systems are suitable to collect high-resolution hyperspectral data for spectral analysis. A dozen or several signature bands can be extracted from the hyperspectral data and can be applied in a portable MSI system for rapid and massive detection.

Several studies developed the spectral band selection or signature-band extraction based on the deep learning models, as listed in Table 1. To improve the computation duration, Zhan et al*.*^19^ eliminated the redundant bands by the distance density. To find the importance of the corresponding bands in a model, Darling et al*.*^20^ applied the Frobenius norm to obtain the value of each row vector delivering a contribution vector by getting the trained weight matrix. Mou et al*.*^21^ proposed an unsupervised deep reinforcement technique for hyperspectral band selection. Elkholy et al*.*^22^ proposed a deep-encoder-based unsupervised hyperspectral band selection method to perform classification. Cai et al*.*^23^ reduced redundant bands with contribution map-based CNN.

**Table 1:** Studies on signature-band extraction or selection with deep learning methods.

Reference	Method	Target
Zhan et al*.*^19^	Occlusion based method	Classification of Indian Pines data sets
Darling et al*.*^20^	Produced contribution matrix by trained weight matrix	Classification of U.S. Army ERDC data sets
Mou et al*.*^21^	Deep reinforcement learning	Classification of Pavia University, Botswana, Indian Pines, and MUUFL Gulfport data sets
Elkholy et al*.*^22^	Unsupervised learning with deep autoencoder unmixing	Classification of Kennedy Space Center data sets
Cai et al*.*^23^	Contribution map-based CNN	Classification of Pavia University and Indian Pines data sets
This study	Absolute mean of the integrated gradients method	Regression on multispectral data sets

The deep learning models with hyperspectral data were successfully used to predict the sugar content of the *Syzygium samarangense* with the MAE of 0.5°Brix in our previous study^24^. The results indicated that the deep learning models with hyperspectral data were beneficial in conducting non-destructive prediction for the sugar content of fruits. If the sugar content can be predicted by several spectral bands, the detection time can be greatly shortened. How to extract the signature bands from hyperspectral data to conduct a rapid and accurate sugar content prediction is a critical issue that is focused in this study. To deal with this issue, this paper presents a new method to extract six signature bands from the deep learning models trained with spectral data. The six signature bands were further utilized to train new deep-learning models to perform the sugar content prediction. The models using six signature bands had the mean absolute errors (MAEs) lower than 0.5°Brix when predicting the sugar content. Thus, the resulting performance of those models using six signature bands could be as good as that of the commercial Brix meters. Furthermore, the performance of the models using the signature bands with different bandwidths in different spectral ranges was evaluated. Firstly, the bandwidth of signature bands can be optimized for sugar content prediction because the bandwidth of spectral bands measured by an MSI system can be adjusted. Secondly, the spectral range of an MSI system is associated with the sensor equipped on this system. For example, the spectral response of a silicon-based complementary metal–oxide–semiconductor sensor with and without an infrared filter covers the visible (VIS) spectrum ranging from 400 to 700 nm and the visible to near-infrared (VISNIR) spectrum ranging from 400 to 1000 nm, respectively. The spectral response of an indium gallium arsenide sensor covers the short-wave infrared (SWIR) ranging from 900 to 2500 nm. Thus, which spectral range and bandwidth of signature bands can be employed to effectively identify the sugar content is a novel exploration in this paper.

The sugar content prediction of *Syzygium samarangense* was taken as an example in this paper. “Materials and data pre-processing” introduces how we collected the hyperspectral data of the peel surface of wax apples and performed the pre-processing of the hyperspectral data. “Proposed methods” addresses the method and procedure of signature-band extraction from hyperspectral data. This section includes three subsections. Firstly, CNN and FNN models were trained and tested with the spectral data of five bandwidths in the four wavelengths, respectively. Secondly, the signature bands were assessed from these well-trained models. Thirdly, new CNN and FNN models were trained with the signature-band data and evaluated on the performance of sugar content prediction, respectively. Thus, “Experimental results” presents the experimental details and results of three subsections in “Proposed methods”. “Conclusions and discussion” provides a comprehensive discussion of the results and highlights the important conclusions and future works drawn from this study.

## Materials and data pre-processing

### Sample preparation

**Figure 1:**
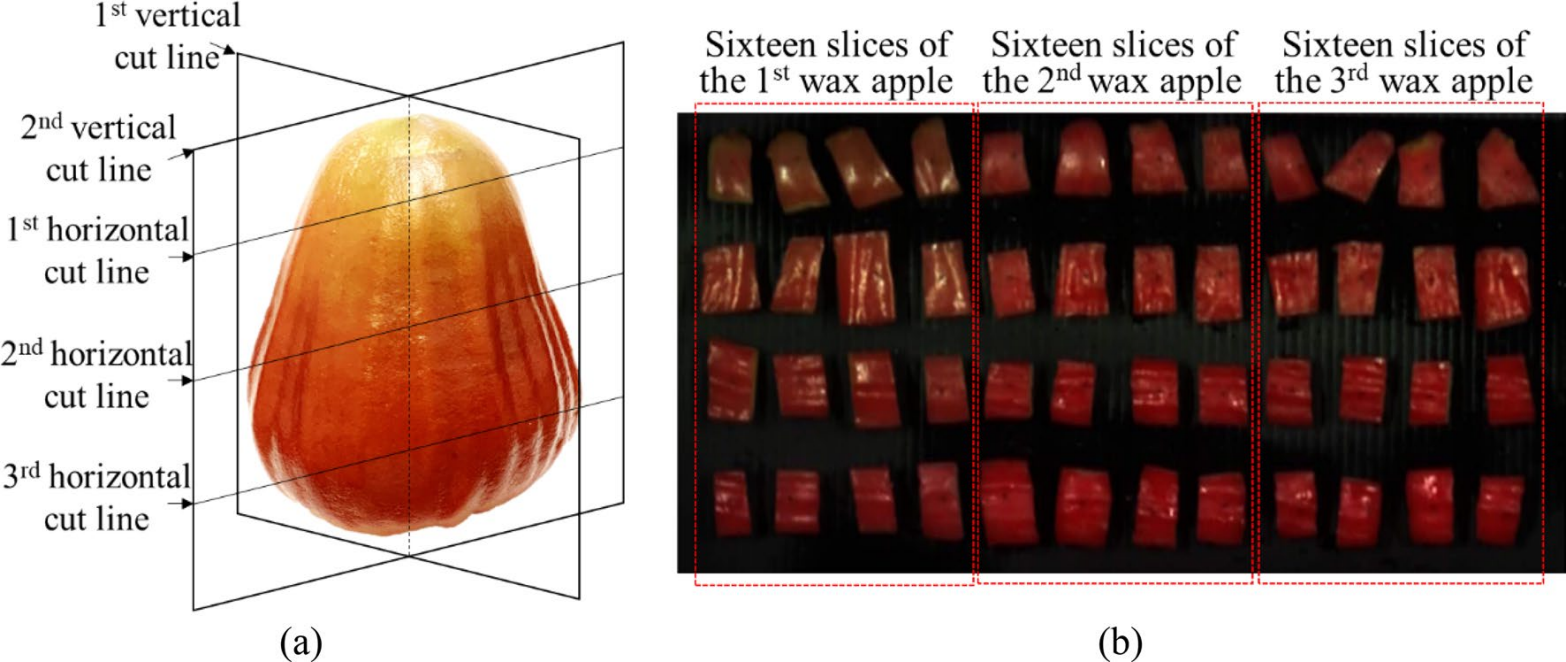
(**a**) An intact wax apple marked with cut lines and (**b**) slices of three wax apples fixed on a flat plate for hyperspectral measurements.

This study takes fruits of wax apple, whose scientific name is *Syzygium samarangense,* as an example of hyperspectral signature-band extraction for sugar content prediction. The species of these apples were Tainung No.3 Sugar Barbie^4^. All data collection procedures were conducted at the Fengshan Tropical Horticultural Experiment Branch (FTHEB), Kaohsiung, Taiwan. 136 wax apples were purchased from Meishan, Fengshan, Liouguei, and Jiadong and refrigerated in a laboratory at FTHEB. The water content of each wax apple needs to keep consistent because the water content affects the spectral reflectance of the wax apples as well as the temperature of wax apples. Thus, refrigerated wax apples were placed in a 25 °C environment and waited for their temperature to return to 25 °C. Afterward, the wax apples were chopped into 16 slices with two vertical and three horizontal cuts, as shown in Fig. 1.

### Hyperspectral data collection

The hyperspectral data collection was conducted in a dark room and performed by the coaxial heterogeneous HSI system^25^. This system can concurrently acquire the VIS and SWIR spectral data of the wavelength ranging from 400 to 1700 nm. The raw hyperspectral image is three-dimensional data (*W*, *L*, Λ), where *W* is image width, *L* is image length, Λ is the number of spectral bands. The spectral data were divided into four wavelength ranges for spectral analysis, including 400–1700 nm, 400–700 nm, 400–1000 nm, and 900–1700 nm where the band numbers of the hyperspectral data in these four spectral ranges are 1367, 575, 1053, and 424, respectively.

### Destructive sugar content measurement

After the hyperspectral data collection, the wax apple slices were squeezed to extract their juice. The sugar contents of these juice were measured by a commercial refractometer ATAGO PAL-1. Organisation Internationale de métrologie légale^26^ recommended that the refractometers can be used to determine the sugar content of fruit juices. The refractometer provides the index of °Brix, which correlates to total soluble solids (TSS) concentration. The TSS of the juice is mainly composed of glucose, fructose, and sucrose. Thus, TSS concentration in juice can represent its sugar content.

### Spectral calibration and denoising

To eliminate the effect of the dark offset and system response, the raw hyperspectral data, *I*_*s*_(*x*, *y*, *λ*), minus dark, *I*_*D*_(*x*, *y*,* λ*), and divided by the spectrum of a standard calibration whiteboard, *I(x*, *y*,* λ*). Thus, the reflectance *R*(*x*, *y*, *λ*) could be derived from Eq. (1), written as

1 $$R\left( {x,y,\lambda_{i} } \right) = \frac{{I_{S} \left( {x,y,\lambda_{i} } \right) - I_{D} (x,y,\lambda_{i} )}}{{I_{w} \left( {x,y,\lambda_{i} } \right) - I_{D} (x,y,\lambda_{i} )}}.$$

The noise of the reflectance data was reduced by adopting a Savitzky–Golay filter, written as

2 $$R_{SGF} (x,y,\lambda_{i} ) = \sum\limits_{k = (1 - w)/2}^{(w - 1)/2} {C_{k} R(x,y,\lambda_{i + k} )} .$$

### Data augmentation

The bottom part of wax apples has the highest sugar content. Thus, the bottom slices were used for sugar content modeling and prediction. A total of 1034 slices were recruited for spectral analysis. The image width and length of the slices are approximately 30–100 pixels and 70–160 pixels, respectively. An area of 20 by 20 pixels in each slice was used. Thus, the hyperspectral cubic data of each slice had the size of 20 × 20 × 1367. These three-dimensional data were the input of the CNN model. For the FNN model, 20 by 20 values in each band were averaged. Thus, the three-dimensional data were transferred to the one-dimension array with the size of 1 × 1367.

Each slice is seen as an individual sample which is measured with a °Brix value. The °Brix of the samples mostly ranged between 8 to 14. The samples were divided into five groups to observe the regression results of sugar content according to their °Brix values. The taste of fruits below the °Brix of 10 is not actually sweet. Thus, the first group contained the samples whose °Brix value was lower than 10. The second, third, and fourth groups contained the samples whose °Brix value was between 10 to 11, 11 to 12, and 12 to 13, respectively. The fifth group contained the samples whose °Brix value was larger than 13. Basically, 218 samples in most of °Brix levels were randomly selected for sugar content regression and divided into training, validation, and test sets, as shown in Table 2. The sample numbers of training, validation, and test sets were 131, 44, and 43, respectively.

**Table 2:** Some statistics of experimental materials, including the mean and standard deviation in each °Brix interval of samples and the sample numbers of training, validation, and test sets in each data type and spectral range. STD denotes the standard deviation of °Brix values.

Data Type	Spectral range (nm)	Size (*W* × *L* × Λ)	Groups			Number of samples			
			°Brix interval	Mean	STD	Train	Valid	Test	Total
3-D data	400–1700	20 × 20 × 261	[00,10]	8.65	0.82	131	44	43	218
			[10,11]	10.44	0.25	97	33	32	162
			[11,12]	11.48	0.23	131	44	43	218
			[12,13]	12.52	0.27	131	44	43	218
			[13,17]	14.01	0.88	131	44	43	218
	400–1000	20 × 20 × 121	[00,10]	8.65,	0.82	131	44	43	218
			[10,11]	10.44	0.25	97	33	32	162
			[11,12]	11.48	0.23	131	44	43	218
			[12,13]	12.52	0.27	131	44	43	218
			[13,17]	14.01	0.88	131	44	43	218
	400–700	20 × 20 × 61	[00,10]	8.65	0.82	97	33	32	218
			[10,11]	10.44	0.25	131	44	43	162
			[11,12]	11.48	0.23	131	44	43	218
			[12,13]	12.52	0.27	131	44	43	218
			[13,17]	14.01	0.88	131	44	43	218
	900–1700	20 × 20 × 161	[00,10]	8.65	0.82	97	33	32	218
			[10,11]	10.44	0.25	131	44	43	162
			[11,12]	11.48	0.23	131	44	43	218
			[12,13]	12.52	0.27	131	44	43	218
			[13,17]	14.01	0.88	131	44	43	218
1-D data	400–1700	1 × 261	[00,10]	8.65	0.82	131	44	43	218
			[10,11]	10.44	0.25	97	33	32	162
			[11,12]	11.48	0.23	131	44	43	218
			[12,13]	12.52	0.27	131	44	43	218
			[13,17]	14.01	0.88	131	44	43	218
	400–1000	1 × 121	[00,10]	8.65	0.82	131	44	43	218
			[10,11]	10.44	0.25	97	33	32	162
			[11,12]	11.48	0.23	131	44	43	218
			[12,13]	12.52	0.27	131	44	43	218
			[13,17]	14.01	0.88	131	44	43	218
	400–700	1 × 61	[00,10]	8.65	0.82	131	44	43	218
			[10,11]	10.4	0.25	97	33	32	162
			[11,12]	11.48	0.23	131	44	43	218
			[12,13]	12.52	0.27	131	44	43	218
			[13,17]	14.01	0.88	131	44	43	218
	900–1700	1 × 161	[00,10]	8.65	0.82	131	44	43	218
			[10,11]	10.44	0.25	97	33	32	162
			[11,12]	11.48	0.23	131	44	43	218
			[12,13]	12.52	0.27	131	44	43	218
			[13,17]	14.01	0.88	131	44	43	218

## Proposed methods

**Figure 2:**
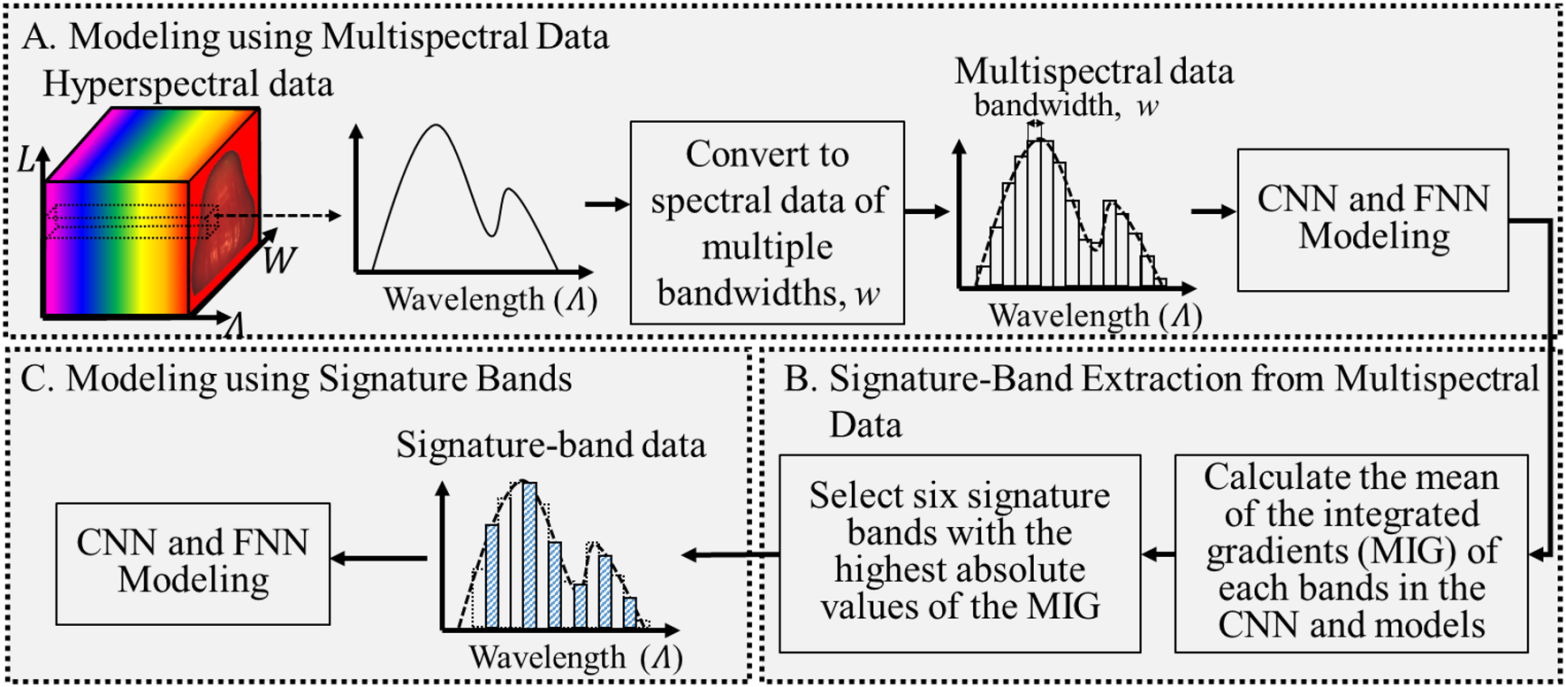
Procedure of the proposed method for signature-band extraction.

This study aims to extract the signature bands from hyperspectral data for rapid sugar content prediction. The bandwidth of the hyperspectral data measured by the coaxial heterogeneous HSI system is nanometer to sub-nanometer. In contrast, the multispectral data measured by an MSI system is approximately greater than 5 nm. As shown in Fig. 2, block A introduces that the hyperspectral data are converted to spectral data with multiple bandwidths to simulate multispectral data. The spectral data are used to create the CNN and FNN-based sugar content prediction models. The models are trained, verified, and tested. Block B illustrates that these models are used to extract the signature bands by the absolute mean of the integrated gradients method. Block C depicts that the CNN-based and FNN-based models are trained, verified, and tested with signature-band data for sugar content prediction.

### Modeling using multispectral data

#### Hyperspectral data conversion

In the coaxial heterogeneous HSI system, the VIS spectrometer has a spectral resolution of ~ 0.5 nm in the wavelength ranging from 400 to 1000 nm; the SWIR spectrometer has a spectral resolution of ~ 2.5 nm in the wavelength ranging from 900 to 1700 nm. The hyperspectral data were converted to spectral data, *R*_*M*_(*x*,*y*,*λ*_c_), which is written as

3 $${\text{R}}_{M} \left( {x,\;y,\;\lambda_{c} } \right) = \frac{1}{{\lambda_{b} - \lambda_{a} }}\left[ {\sum\limits_{i = a}^{b} {{\text{R}}_{SGF} \left( {x,y,\lambda_{i} } \right) \times \left( {\lambda_{i + 1} - \lambda_{i} } \right)} } \right]{, }\,[\lambda_{a} ,\lambda_{b} ] \in \left[ {\lambda_{c} - \frac{w}{2},\lambda_{c} + \frac{w}{2}} \right]{ ,}$$

where R_M_(*x*,*y*,*λ*_c_) is the mean of the integrated intensity of the central band,* λ*_c_; the central band *λ*_c_ ranges from 400 to 1700 nm with an interval of bandwidth, *w*.

A total of six sets of spectral data were converted from hyperspectral data according to the six bandwidths, $w$, of ± 2.5 nm, ± 5 nm, ± 7.5 nm, ± 10 nm, ± 12.5 nm, and ± 15 nm. Each set was grouped into four subsets based on the spectral range of 400–1700 nm, 400–1000 nm, 400–700 nm, and 900–1700 nm, as shown in Table 3.

**Table 3:** Band numbers of hyperspectral data and multispectral data with six bandwidths in four spectral ranges.

Data type	Bandwidth (nm)	Band numbers			
		400–1700 nm	400–1000 nm	400–700 nm	900–1700 nm
Hyperspectral data	± 0.25 ~ ± 1.25	1367	1053	575	424
Multispectral data	± 2.5	261	121	61	161
	± 5	130	60	30	80
	± 7.5	87	40	20	53
	± 10	65	30	15	40
	± 12.5	52	24	12	32
	± 15	43	20	10	27

We apply the exponential learning rate decay method in Eq. (4) with the initial learning rate *lr* of the CNN and FNN models. The exponential learning rate decay method is written as

4 $$lr = lr \times \exp^{( - k \times j)} ,$$

where *lr* is the learning rate, and $k$ is the decaying rate; *j* is the iteration number of the epoch. The performance of the CNN and FNN models for sugar content prediction was evaluated by the mean absolute error, MAE, which is defined as

5 $${\text{MAE}} = \frac{{\sum\nolimits_{i = 1}^{n} {\left| {y_{i} - \hat{y}_{i} } \right|} }}{n},$$

where *y*_*i*_ is the prediction value of the sugar content, $\widehat{y}$ is the true value of the sugar content, and *n* is the sample size. The goodness-of-fit of the CNN and FNN models was assessed by the R-squared value which is defined by

6 $${\text{R}}^{2} = 1 - \frac{{\sum\nolimits_{i = 1}^{n} {\left( {y_{i} - \hat{y}_{i} } \right)^{2} } }}{{\sum\nolimits_{i = 1}^{n} {\left( {y_{i} - \overline{y}} \right)^{2} } }},$$

where $\overline{y }$ is the mean of the prediction values. An R^2^ value of zero indicates that the regression of a model doesn’t fit the data properly, while an R^2^ value of 1 indicates that the regression of the model fits perfectly.

#### CNN modeling and verification using multispectral data

**Figure 3:**
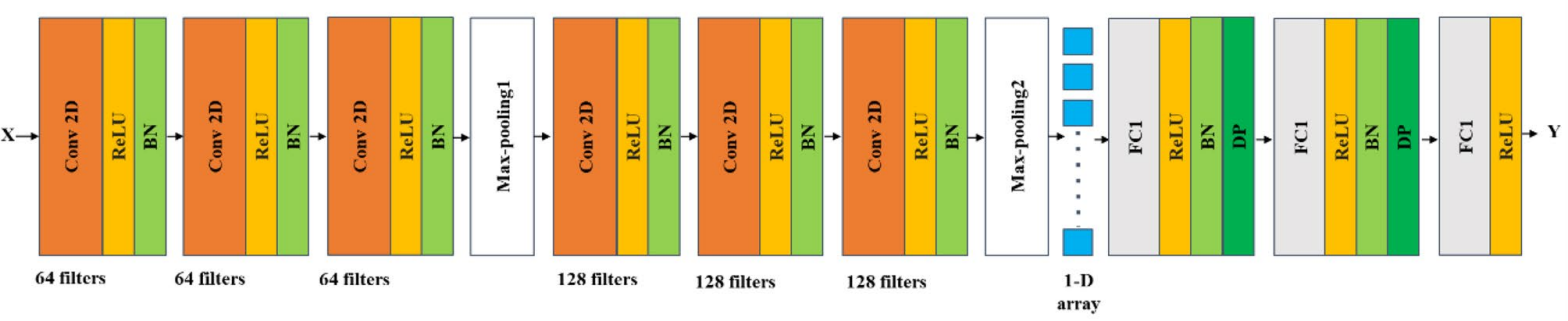
The proposed convolution neural network model with the input of three-dimensional spectral data for sugar content prediction.

The architecture of the CNN model for modeling multispectral data is shown in Fig. 3. The input of this model is three-dimensional data (*W*, *L*, Λ). The number of bands, Λ, was tens to hundreds. The proposed CNN model is composed of 2D convolution layers (Conv2D) with L2-norm regularization, Batch Normalization layers (BN), Dropout layers (DP), and Fully-Connected layer (FC). All the neurons were activated by the Rectified Linear Unit (ReLU), and the loss is measured by the Root Mean Squared Logarithmic Error (RMSLE).

#### FNN modeling and verification using multispectral data

**Figure 4:**
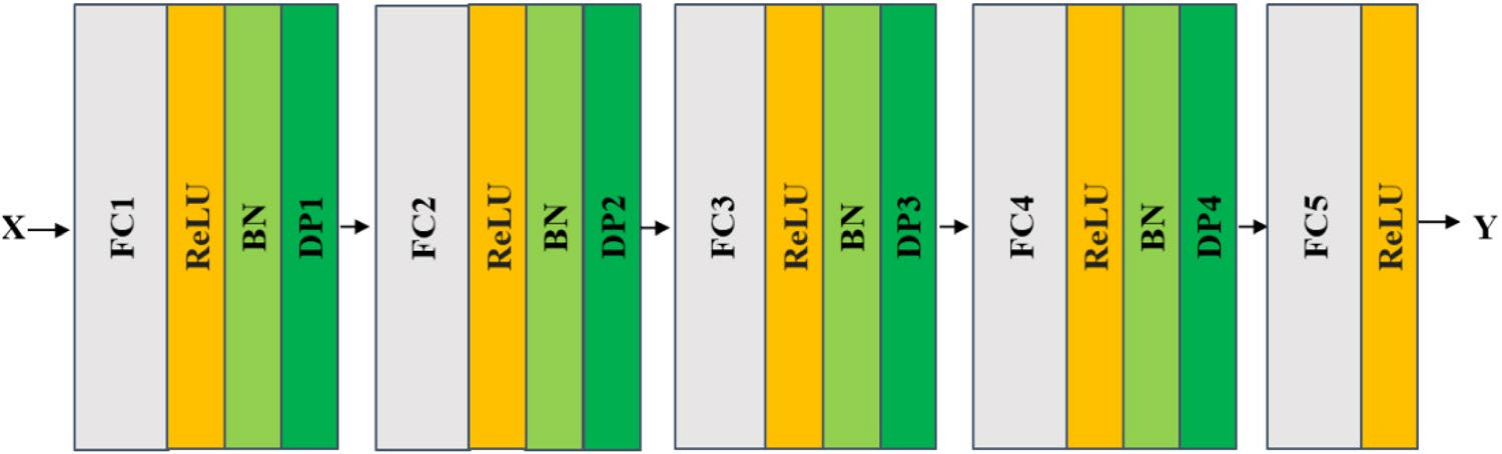
The proposed feedforward neural network model with the input of one-dimensional spectral data for sugar content prediction.

The architecture of the FNN model for modeling multispectral data is shown in Fig. 4. The input of the model is one-dimensional data with a size for tens to hundreds of bands. The CNN model consists of 4 FC-ReLU-BN-DP layers. The final output Y is the sugar content prediction result of the model. The FNN model is similar to the proposed CNN model. The weights with the smallest RMSLE are recorded for the test datasets.

### Signature-band extraction from multispectral data

According to the integrated gradient defined in Ref.^27^, the mean of the integrated gradients (MIG), derived from $S$ samples, is written as

7 $${\text{MIG}} = \frac{1}{S} \times \frac{1}{M} \times \sum\limits_{i = 0}^{S} {\sum\limits_{k = 1}^{M} {\frac{{\partial F\left[ {R_{i}^{\prime } + \frac{k}{M} \times (R_{i} - R_{i}^{\prime } )} \right]}}{{\partial r_{i} }}} } ,$$

where *R* is the input data, and *M* is the number of intervals along the path from the baseline from *R*^′^ to *R*. Most deep learning frameworks could efficiently perform the calculation of the gradient.

8 $${\text{Score}} (\lambda_{C} ) = \left| {\left. {\frac{1}{W \times L}\sum\limits_{W} {\sum\limits_{L} {{\text{MIG}} \left( {W,L,\lambda_{C} } \right)} } } \right|} \right..$$

The MIG of the CNN models is a three-dimensional matrix. The three-dimensional MIG is averaged along the width, *W*, and length, *L*. Afterward, a one-dimensional array, Score, with the data points corresponding to the bands, *λ*_c_, is obtained, as defined in Eq. (8). The MIG of the FNN models is a one-dimensional matrix. The Score of the FNN is the absolute of the MIG. The Score represents the contribution score of bands in a deep learning model. The significance of a band in a model is positively correlated to the Score of this band.

The band with the first highest Score is selected for the first signature band. The second signature band is selected according to the band which has the second highest Score and is out of the ± 20 nm range of the first selected band, and so on.

### Modeling using signature bands

The hyperspectral data of the signature bands are resampled into the spectral data with the bandwidths of ± 25 nm, ± 20 nm, ± 15 nm, ± 10 nm, and ± 5 nm. The spectral data are used to train new CNN and FNN models for sugar content prediction. The input data of the CNN and FNN models were three-dimensional hyperspectral and one-dimensional spectral data of the signature bands, respectively. The band number of a signature-band set is six.

**Figure 5:**
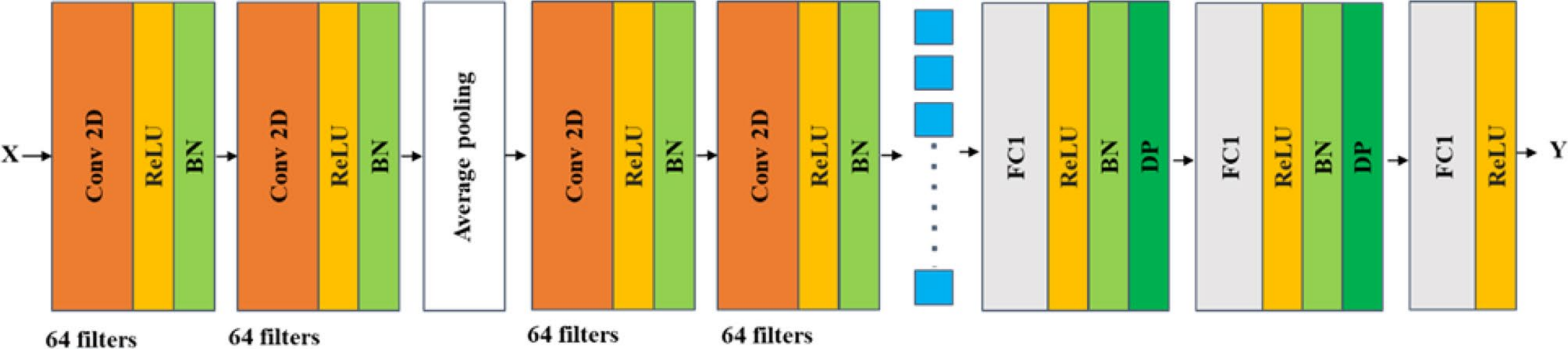
The proposed convolution neural network model with the input of three-dimensional multispectral data for sugar content prediction.

The architecture of the CNN model for modeling resampled spectral data is shown in Fig. 5. The input of this model is three-dimensional data (*W*, *L*, Λ). The number of bands, Λ, was six. The CNN model consists of two Conv-ReLu-BN layers with 64 kernel filters, two Conv-ReLU-BN layers with 64 kernel filters, one Average Pooling layer, two FC-ReLU-BN-DP layers, and the output layers with the rectified linear unit (ReLU) as the activation function. Because the number of bands, Λ, diminished to 6, some layers were reduced and the Average Pooling instead of Max-Pooling for the smaller size resampled data was used.

**Figure 6:**
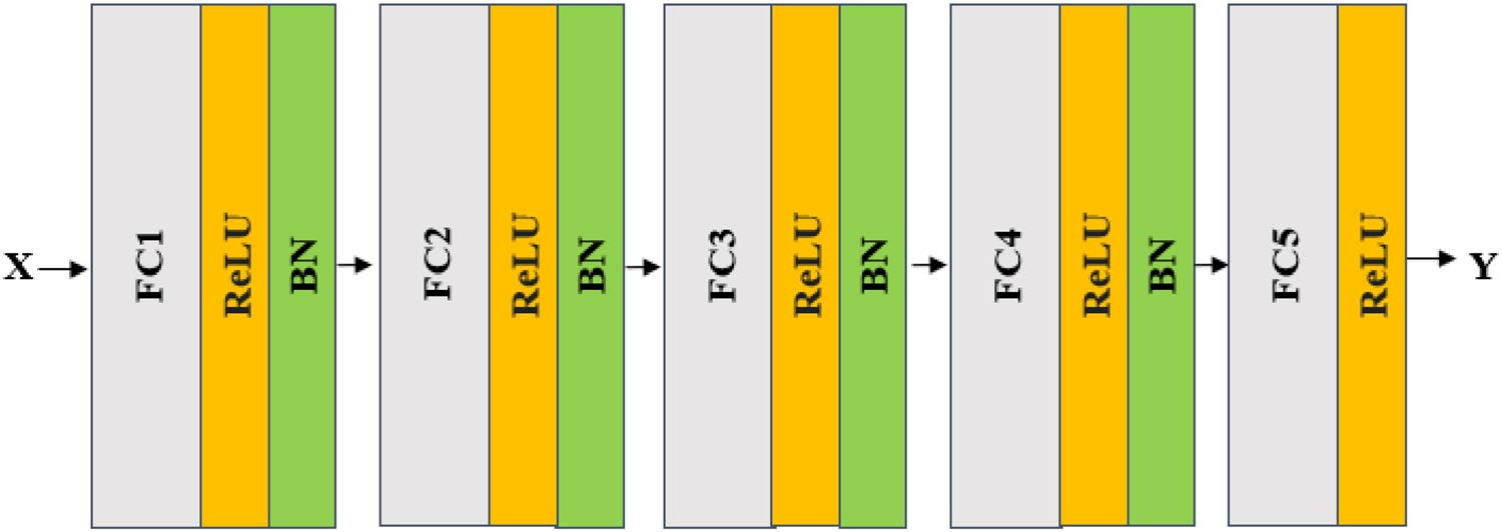
The proposed feedforward neural network model with the input of one-dimensional signature-band data for sugar content prediction.

The architecture of the FNN model for modeling multispectral data is shown in Fig. 6. The input of the model is one-dimensional data with a size of six bands. The FNN model consists of 4 FC-ReLU-BN layers. The final output Y is the sugar content prediction result of the model. The FNN model is similar to the proposed CNN model; the weights with the smallest RMSLE are recorded for the test datasets.

## Experimental results

### Results of the modeling using multispectral data

Modeling with the spectral data in different wavelength ranges might have various results; thus, the spectral range was divided into four spectral ranges, including VIS (400–700 nm), VISNIR (400–1000 nm), SWIR (900–1700 nm), and VISWIR (400–1700 nm). The hyperparameters of the CNN and FNN models are shown in Tables 4 and 5. The CNN and FNN models using spectral data of six bandwidths assumed the same ten hyperparameters and nine hyperparameters, respectively. The exponential learning rate decay method is applied with the initial learning rate, and the decaying rate *k* is 1 × 10^–6^. Adam optimizer was also applied. In the CNN model, the regularization parameter is 1 × 10^–10^ for L2 regularization.

**Table 4:** Selected hyperparameters of the convolution neural network model with multispectral data.

Spectral range	400–1700 nm	400–1000 nm	400–700 nm	900–1700 nm
Conv. kernel size	1 × 5	1 × 2	1 × 2	1 × 5
MaxPooling1 size	1 × 2	1 × 2	2 × 1	2 × 2
MaxPolling2 size	1 × 2	2 × 1	2 × 1	2 × 2
FC1 units	2048	1024	1024	1024
FC2 units	512	256	512	512
FC3 units	1	1	1	1
Batch size	32	64	128	32
Dropout rate	0.3	0.3	0.2	0.1
Learning rate	0.0006	0.0006	0.0006	0.001

**Table 5:** Selected hyperparameters of the feedforward neural network model with multispectral data.

Spectral range	400–1700 nm	400–1000 nm	400–700 nm	900–1700 nm
FC1 units	256	256	256	256
FC2 units	128	128	128	128
FC3 units	64	64	64	64
FC4 units	32	32	32	32
FC5 units	1	1	1	1
Batch size	32	64	128	64
Dropout rate	0.3	0.3	0.3	0.3
Learning rate	0.001	0.001	0.001	0.001

The prediction results of the CNN and FNN models using multispectral data with six bandwidths in four spectral ranges are shown in Table 6. The CNN and FNN models using the spectral data of ± 2.5 nm bandwidth had the worst prediction performance compared to those using the spectral data with the other bandwidths. Furthermore, the CNN and FNN models using the spectra in the SWIR spectral range had the worst prediction performance compared to those using the spectra in the other spectral ranges. Thus, the spectral data of ± 2.5 nm bandwidth and the spectra in the SWIR spectral range were excluded from the signature-band extraction.

**Table 6:** Sugar content prediction results of the convolution neural network and feedforward neural network models using the multispectral data of six bandwidths in four spectral ranges. MAE denotes the mean absolute error of the predictions. R^2^ denotes the R-squared values of the models. Bold values denote the best results of the sugar content prediction in the CNN and FNN models.

Model	Spectral range	MAE and R^2^					
		± 2.5 nm	± 5 nm	± 7.5 nm	± 10 nm	± 12.5 nm	± 15 nm
CNN	400–1700 nm	0.723	0.475	0.437	0.466	**0.400**	0.484
		0.702	0.864	0.886	0.871	0.901	0.849
	400–1000 nm	0.679	0.619	0.489	0.536	0.511	0.501
		0.708	0.278	0.868	0.840	0.874	0.860
	400–700 nm	0.642	0.523	0.474	0.570	0.482	0.509
		0.737	0.800	0.874	0.758	0.848	0.843
	900–1700 nm	0.739	0.857	0.870	0.923	0.802	0.797
		0.619	0.571	0.577	0.511	0.578	0.603
FNN	400–1700 nm	0.603	0.540	0.507	0.478	0.451	0.519
		0.825	0.864	0.883	0.896	0.903	0.868
	400–1000 nm	0.614	0.465	0.459	0.491	0.484	**0.408**
		0.811	0.896	0.898	0.881	0.890	0.920
	400–700 nm	0.532	0.527	0.450	0.434	0.491	0.487
		0.857	0.879	0.898	0.908	0.887	0.881
	900–1700 nm	0.771	0.692	0.802	0.776	0.869	0.886
		0.724	0.770	0.705	0.718	0.671	0.653

### Results of signature-band extraction

**Figure 7:**
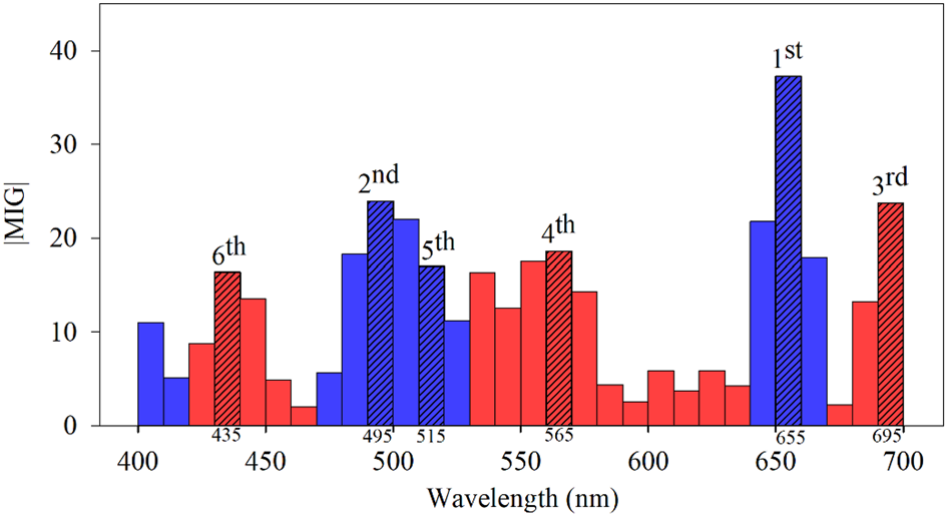
Absolute mean of the integrated gradients of the feedforward neural network model with a bandwidth of ± 5 nm in a spectral range of 400–700 nm. The top six Score ranked bands are shown by the bars with slash texture and selected as the signature bands, as listed in Table 7. The blue bars represent that the MIG value of the bands is positive. The red bars represent that the MIG value of the bands is negative.

Thirty deep learning models were trained by the spectral data with five bandwidths in three spectral ranges. The five bandwidths included ± 5 nm, ± 7.5 nm, ± 10 nm, ± 12.5 nm, and ± 15 nm, and the three spectral ranges were 400–700 nm, 400–1000 nm, and 400–1700 nm. The contribution of the bands used by each model was assessed by the Score, which is the absolute of the MIG, according to Eqs. (7) and (8). The top six spectral bands with the highest Score values of each model are listed in Tables 7, 8, 9, 10 and 11. Each entry in these tables includes the spectral band and its MIG value. The absolute MIG values of the FNN model using spectral data with a bandwidth of ± 5 nm in a spectral range of 400–700 nm are shown in Fig. 7. The six signature bands in this FNN model were selected according to the criteria introduced in sub-section B of section III and marked in the bars with a slash texture.

**Table 7:** Top six Score ranked spectral bands associated with the convolution neural network and feedforward neural network models using the spectral data with a bandwidth of ± 5 nm in three spectral ranges. The Socre is the absolute mean of the integrated gradients.

Model	Spectral range	Bands (nm) and their mean of the integrated gradients					
		1st	2nd	3rd	4th	5th	6th
CNN	400–1700 nm	565 nm	585 nm	515 nm	545 nm	745 nm	695 nm
		− 0.056	− 0.030	0.029	− 0.022	0.020	− 0.019
	400–1000 nm	685 nm	565 nm	655 nm	545 nm	495 nm	475 nm
		− 0.083	− 0.076	0.073	− 0.054	− 0.053	0.053
	400–700 nm	485 nm	445 nm	625 nm	545 nm	495 nm	475 nm
		0.111	0.093	− 0.071	0.066	− 0.063	0.058
FNN	400–1700 nm	565 nm	655 nm	505 nm	1155 nm	485 nm	585 nm
		− 14.74	14.085	12.355	11.536	9.276	− 8.535
	400–1000 nm	575 nm	655 nm	505 nm	555 nm	485 nm	595 nm
		− 14.289	13.686	12.766	− 11.332	9.22	− 7.660
	400–700 nm	655 nm	495 nm	695 nm	565 nm	515 nm	435 nm
		37.260	23.970	− 23.750	− 18.619	16.998	− 16.412

**Table 8:** Top six Score ranked spectral bands associated with the convolution neural network and feedforward neural network models using the spectral data with a bandwidth of ± 7.5 nm in three spectral ranges. The Socre is the absolute mean of the integrated gradients.

Model	Spectral range	Bands (nm) and their mean of the integrated gradients					
		1st	2nd	3rd	4th	5th	6th
CNN	400–1700 nm	587.5 nm	542.5 nm	677.5 nm	647.5 nm	992.5 nm	737.5 nm
		− 0.134	0.089	0.076	0.056	0.043	− 0.038
	400–1000 nm	512.5 nm	452.5 nm	602.5 nm	572.5 nm	692.5 nm	662.5 nm
		0.270	− 0.187	− 0.169	− 0.167	− 0.140	0.132
	400–700 nm	572.5 nm	497.5 nm	452.5 nm	617.5 nm	662.5 nm	527.5 nm
		− 0.090	0.073	− 0.062	0.030	0.024	0.023
FNN	400–1700 nm	572.5 nm	662.5 nm	497.5 nm	1172.5 nm	707.5 nm	602.5 nm
		− 19.366	14.214	13.285	12.81	− 9.030	− 8.708
	400–1000 nm	575.5 nm	497.5 nm	662.5 nm	707.5 nm	962.5 nm	527.5 nm
		− 21.109	− 16.406	− 12.557	− 8.242	− 7.978	6.651
	400–700 nm	662.5 nm	692.5 nm	557.5 nm	497.5 nm	437.5 nm	632.5 nm
		42.964	− 36.237	− 31.850	28.393	− 18.123	− 10.25

**Table 9:** Top six Score ranked spectral bands associated with the convolution neural network and feedforward neural network models using the spectral data with a bandwidth of ± 10 nm in three spectral ranges. The Socre is the absolute mean of the integrated gradients.

Model	Spectral range	Bands (nm) and their mean of the integrated gradients					
		1st	2nd	3rd	4th	5th	6th
CNN	400–1700 nm	570 nm	590 nm	510 nm	530 nm	650 nm	630 nm
		− 0.079	− 0.074	0.064	0.056	0.045	0.036
	400–1000 nm	530 nm	550 nm	590 nm	710 nm	450 nm	510 nm
		0.092	0.087	− 0.057	− 0.053	− 0.038	− 0.035
	400–700 nm	510 nm	530 nm	610 nm	490 nm	590 nm	450 nm
		0.072	0.035	− 0.034	0.033	− 0.032	− 0.027
FNN	400–1700 nm	570 nm	510 nm	590 nm	1190 nm	1170 nm	490 nm
		− 23.476	16.677	− 16.253	− 16.205	14.884	14.346
	400–1000 nm	570 nm	590 nm	510 nm	650 nm	670 nm	490 nm
		− 25.192	− 20.786	18.080	13.278	12.671	11.789
	400–700 nm	490 nm	670 nm	510 nm	550 nm	690 nm	570 nm
		46.826	43.590	40.398	− 38.985	− 36.393	− 32.428

**Table 10:** Top six Score ranked spectral bands associated with the convolution neural network and feedforward neural network models using the spectral data with a bandwidth of ± 12.5 nm in three spectral ranges. The Socre is the absolute mean of the integrated gradients.

Model	Spectral range	Bands (nm) and their mean of the integrated gradients					
		1st	2nd	3rd	4th	5th	6th
CNN	400–1700 nm	587.5 nm	662.5 nm	512.5 nm	637.5 nm	712.5 nm	562.5 nm
		− 0.069	0.060	0.046	0.041	− 0.041	− 0.034
	400–1000 nm	512.5 nm	662.5 nm	562.5 nm	587.5 nm	537.5 nm	687.5 nm
		0.163	0.134	− 0.102	− 0.079	− 0.039	− 0.039
	400–700 nm	512.5 nm	587.5 nm	562.5 nm	662.5 nm	612.5 nm	462.5 nm
		0.129	− 0.123	− 0.099	0.078	0.077	− 0.044
FNN	400–1700 nm	587.5 nm	562.5 nm	662.5 nm	512.5 nm	1187.5 nm	712.5 nm
		− 21.876	− 21.629	18.925	17.093	13.427	− 12.145
	400–1000 nm	587.5 nm	562.5 nm	662.5 nm	512.5 nm	712.5 nm	487.5 nm
		− 32.426	− 29.572	29.164	28.083	− 15.352	14.503
	400–700 nm	662.5 nm	562.5 nm	487.5 nm	512.5 nm	587.5 nm	687.5 nm
		55.080	− 41.580	40.196	36.291	− 28.255	22.905

**Table 11:** Top six Score ranked spectral bands associated with the convolution neural network and feedforward neural network models using the spectral data with a bandwidth of ± 15 nm in three spectral ranges. The Socre is the absolute mean of the integrated gradients.

Model	Spectral range	Bands (nm) and their mean of the integrated gradients					
		1st	2nd	3rd	4th	5th	6th
CNN	400–1700 nm	595 nm	655 nm	685 nm	535 nm	565 nm	895 nm
		− 0.041	0.031	0.027	0.019	− 0.018	− 0.018
	400–1000 nm	595 nm	565 nm	655 nm	535 nm	625 nm	505 nm
		− 0.084	− 0.082	0.055	0.053	0.028	0.024
	400–700 nm	505nm	565 nm	475 nm	535 nm	595 nm	625 nm
		0.295	− 0.221	− 0.137	0.077	− 0.067	0.036
FNN	400–1700 nm	565 nm	595 nm	505 nm	1195 nm	655 nm	715 nm
		− 23.844	− 21.856	21.712	16.830	16.287	− 14.651
	400–1000 nm	505 nm	595 nm	565 nm	655 nm	715 nm	895 nm
		34.878	− 33.987	− 30.594	27.233	− 19.088	12.860
	400–700 nm	505 nm	565 nm	655 nm	595 nm	445 nm	625 nm
		58.007	− 46.255	37.925	− 23.789	− 20.799	− 17.332

### Results of modeling using signature bands

Each model trained by the spectral data with a bandwidth in a spectral range had one set of the six signature bands with the six highest absolute MIGs, as listed in Tables 7, 8, 9, 10 and 11. Thus, a total of thirty sets of spectral bands associated with the bandwidths of ± 25 nm, ± 20 nm, ± 15 nm, ± 10 nm, and ± 5 nm in three spectral ranges of 400–1700 nm, 400–1000 nm, and 400–700 nm were selected as signature bands. The performance of the CNN and FNN models trained using these thirty sets of signature bands was further evaluated. The eight hyperparameters of the CNN and FNN models trained by these signature bands with the lowest MAE in each spectral range are shown in Tables 12 and 13, respectively. The exponential learning rate decay method is applied with the initial learning rate, and the decaying rate k is 1 × 10^–6^. Adam optimizer was also applied. In the CNN model, the regularization parameter is 1 × 10^–10^ for L2 regularization.

**Table 12:** Selected hyperparameters of the convolution neural network models using the signature bands with the lowest prediction error in each spectral range.

Spectral range (nm)	400–1700	400–1000	400–700
Bandwidth (nm)	± 10	± 7.5	± 12.5
Conv. kernel size	4 × 1	4 × 1	4 × 1
Avg. pooling size	2 × 2	2 × 2	1 × 4
FC1 units	1024	1024	1024
FC2 units	512	512	512
FC3 units	1	1	1
Batch size	16	128	64
Learning rate	0.0004	0.0008	0.0004

**Table 13:** Selected hyperparameters of the feedforward neural network models using the signature bands that had the lowest prediction error in each spectral range.

Spectral range (nm)	400–1700	400–1000	400–700
Bandwidth (nm)	± 10	± 12.5	± 12.5
FC1 units	512	512	512
FC2 units	256	256	256
FC3 units	128	128	128
FC4 units	64	64	64
FC5 units	1	1	1
Batch size	64	128	128
Learning rate	0.0008	0.0008	0.0008

The sugar content prediction results of the CNN and FNN models using the signature bands are shown in Table 14. The FNN model using the VISWIR signature bands with a bandwidth of ± 12.5 nm had a minimum MAE of 0.390° Brix compared to the other FNN models. The CNN model using the VISWIR signature bands with a bandwidth of ± 10 nm had the lowest MAE of 0.549° Brix compared to the other CNN models. The MAEs of the FNN models were significantly lower than those of the CNN models.

**Table 14:** Sugar content prediction errors of the convolution neural network and feedforward neural network models using the signature bands with the bandwidths of ± 5 nm, ± 7.5 nm, ± 10 nm, ± 12.5 nm, and ± 15 nm in three spectral ranges of 400–700 nm, 400–1000 nm, and 400–1700 nm. MAE denotes the mean absolute error of the predictions. R^2^ denotes the R-squared values of the models. Bold values denote the best results of the sugar content prediction in the CNN and FNN models.

Model	Spectral range	MAE and R^2^					
		± 5 nm	± 7.5 nm	± 10 nm	± 12.5 nm	± 15 nm	Average of MAEs in each row
CNN	400–1700 nm	0.578	0.588	**0.549**	0.573	0.594	0.576
		0.794	0.820	0.811	0.789	0.774	
	400–1000 nm	0.567	0.596	0.634	0.606	0.569	0.585
		0.828	0.783	0.794	0.800	0.812	
	400–700 nm	0.551	0.645	0.582	0.661	0.580	0.604
		0.841	0.785	0.803	0.758	0.849	
FNN	400–1700 nm	0.469	0.498	0.470	**0.390**	0.434	0.452
		0.885	0.866	0.894	0.916	0.893	
	400–1000 nm	0.518	0.418	0.497	0.503	0.447	0.477
		0.860	0.889	0.886	0.876	0.899	
	400–700 nm	0.493	0.451	0.478	0.393	0.465	0.456
		0.877	0.888	0.884	0.903	0.881	
	Average of MAEs in each column	0.529	0.533	0.535	0.521	0.515	

## Conclusions and discussion

This study proposes a signature-band extraction method using the absolute mean of the integrated gradients score to extract signature bands from sugar-content deep-learning models with the input of spectral data. Firstly, the hyperspectral data with bandwidths lower than 2.5 nm were converted to spectral data with bandwidths of ± 2.5 nm, ± 5 nm, ± 7.5 nm, ± 10 nm, ± 12.5 nm, and ± 15 nm. The spectral data were used to train and verify CNN and FNN models. The CNN model using the VISWIR spectral data with a bandwidth of ± 12.5 nm had a minimum MAE of 0.400°Brix compared to that of the other CNN models. Furthermore, the FNN model using the VISNIR spectral data with a bandwidth of ± 15 nm had a minimum MAE of 0.408°Brix compared to the other FNN models.

The absolute MIG method was used to extract signature bands from the CNN and FNN models. The MIG of the deep learning model corresponding to an input of spectral bands can be positive or negative. The MIG implies that the input bands have either positively or negatively correlated to the output of the model. The six spectral bands with the highest positive absolute MIGs were considered to be signature bands. However, the FNN and CNN models trained by these signature bands didn’t have lower prediction errors compared to the models trained by the six signature bands with the highest absolute MIGs. Thus, this study finds the signature bands with the highest absolute MIGs instead of finding the signature bands with the highest positive MIGs. The central wavelength of the top six bands selected from the CNN models in the VISWIR range was not over 1000 nm (Tables 7, 8, 9, 10 and 11). Only one of each six bands over 1000 nm was chosen from the FNN models in the VISWIR range. The results imply that the signature bands in our deep learning models for predicting the sugar content of the wax apples are significantly located in the VIS range. This finding seems to show that the VIS spectrum of the appearance of the wax apples might have correlated considerably with the sugar content of the wax apples.

Thirty sets of six signature bands of spectral data in five bandwidths were used to train the CNN and FNN models for sugar content prediction. The input data of the CNN and FNN models were three-dimension and one-dimension, respectively. The minimum MAE of the CNN and FNN models using six signature bands were 0.549°Brix and 0.390°Brix. Both results were less than 0.55°Brix; and close to or better than that of the CNN and FNN models using hundreds of spectral bands or thousands of spectral bands. The performance of the FNN models was almost better than that of the CNN models in the three spectral ranges of 400–1700 nm, 400–1000 nm, and 400–700 nm. The correlation between two adjacent bands is reduced in spectral data compared to hyperspectral data; the CNN model tends to find the correlation between two spectral bands; this might be why the performance of the CNN model was worse than that of the FNN model. The results reveal that the FNN model with one-dimensional input data might perform better on the sugar content prediction than the CNN model with three-dimensional input data. Furthermore, the CNN and the FNN models using only six signature bands have a high potential to predict the sugar content of wax apples. These six signature bands could be used in an MSI system to non-destructively and rapidly predict the sugar content of the wax apples in the future.

The spectral data was not overlapped in the spectrum when it was converted from the hyperspectral data in this study. However, the spectrum of each spectral band could overlap. Spectral data with overlapped spectrums could be considered for signature-band extraction. Furthermore, the spectral bands used in an MSI system could have different bandwidths. Thus, the signature bands might be chosen from the bands with different bandwidths. These two variables greatly increase the complexity of the band extraction. In the future, an artificial intelligence model may perform signature-band extraction from spectral data with different bandwidths or overlapped spectrums.

## Data Availability

The datasets used and analyzed during the current study are available from the corresponding author upon reasonable request.

## References

[1] Directorate-General of Budget, Accounting, and Statistics, Executive Yuan, Taiwan. Quantity and Value of Farm Products (2023). https://eng.coa.gov.tw/upload/files/eng_web_structure/2505686/ZA_ZA01-4_110.pdf. Accessed: April 26, 2023.

[2] Directorate-General of Budget, Accounting, and Statistics, Executive Yuan, Taiwan. Agricultural Statistics Inquiry (2023). https://agrstat.coa.gov.tw/sdweb/public/trade/TradeCoa.aspx. Accessed: April 26, 2023.

[3] Shu ZH, Tzong SL, Jung ML, Chi CH, Der NW, Hsiao HP (2007). The industry and progress review on the cultivation and physiology of Wax Apple–with special reference to ‘Pink’ variety. Asian Aust. J. Plant Sci. Biotechnol..

[4] Huang, C.-C. New species of wax apple—Tainung No.3 Sugar Barbie. In *Special Publication of Taiwan Agriculture Research Institute Council of Agriculture*, vol. 106, 114–116 (2016).

[5] Bioucas-Dias JM, Plaza A, Camps-Valls G, Scheunders P, Nasrabadi N, Chanussot J (2013). Hyperspectral remote sensing data analysis and future challenges. IEEE Geosci. Remote Sens. Mag..

[6] Akhtar N, Mian A (2017). Nonparametric coupled Bayesian dictionary and classifier learning for hyperspectral classification. IEEE Trans. Neural Netw. Learn. Syst..

[7] Zhong P, Wang R (2014). Jointly learning the hybrid CRF and MLR model for simultaneous denoising and classification of hyperspectral imagery. IEEE Trans. Neural Netw. Learn. Syst..

[8] Peiris KHS, Dull GG, Leffler RG, Kays SJ (1998). Near-infrared spectrometric method for non-destructive determination of soluble solids content of peaches. J. Am. Soc. Hortic. Sci..

[9] Mendoza F, Lu R, Ariana D, Cen H, Bailey B (2011). Integrated spectral and image analysis of hyperspectral scattering data for prediction of apple fruit firmness and soluble solids content. Postharvest Biol. Technol..

[10] Liang PS, Haff RP, Hua SST, Munyaneza JE, Mustafa T, Sarreal SBL (2018). Non-destructive detection of zebra chip disease in potatoes using near-infrared spectroscopy. Biosyst. Eng..

[11] Kemps B, Leon L, Best S (2010). Assessment of the quality parameters in grapes using VIS/NIR spectroscopy. Biosyst. Eng..

[12] Chuang YK, Chen S, Lo YM, Tsai CY, Yang IC, Chen YL, Pan PJ, Chen CC (2012). Integration of independent component analysis with near infrared spectroscopy for rapid quantification of sugar content in wax jambu (*Syzygium samarangense* Merrill & Perry). J. Food Drug Anal..

[13] Viegas TR, Mata AL, Duarte MM, Lima KM (2016). Determination of quality attributes in wax jambu fruit using NIRS and PLS. Food Chem..

[14] Gao Z, Shao Y, Xuan G, Wang Y, Liu Y, Han X (2020). Real-time hyperspectral imaging for the in-field estimation of strawberry ripeness with deep learning. Artif. Intell. Agric..

[15] Itakura K, Saito Y, Suzuki T, Kondo N, Hosoi F (2019). Estimation of citrus maturity with fluorescence spectroscopy using deep learning. Horticulturae.

[16] Tu S, Pang J, Liu H, Zhuang N, Chen Y, Zheng C, Wan H, Xue Y (2020). Passion fruit detection and counting based on multiple scale faster R-CNN using RGB-D images. Precis. Agric..

[17] Fajardo M, Whelan BM (2021). Within-farm wheat yield forecasting incorporating off-farm information. Precis. Agric..

[18] Marani R, Milella A, Petitti A, Reina G (2021). Deep neural networks for grape bunch segmentation in natural images from a consumer-grade camera. Precis. Agric..

[19] Zhan Y, Hu D, Xing H, Yu X (2017). Hyperspectral band selection based on deep convolutional neural network and distance density. IEEE Geosci. Remote Sens. Lett..

[20] Darling PC (2021). Neural network-based band selection on hyperspectral imagery. Artif. Intell. Mach. Learn. Multi-Domain Oper. Appl. III.

[21] Mou L, Saha S, Hua Y, Bovolo F, Bruzzone L, Zhu XX (2021). Deep reinforcement learning for band selection in hyperspectral image classification. IEEE Trans. Geosci. Remote Sens..

[22] Elkholy MM, Mostafa MS, Ebeid HM, Tolba M (2021). Unsupervised hyperspectral band selection with deep autoencoder unmixing. Int. J. Image Data Fus..

[23] Cai, R., Yuan, Y., and Lu, X. Hyperspectral band selection with convolutional neural network. In *Chinese Conference on Pattern Recognition and Computer Vision*, 396–408 (Springer, 2021).

[24] Chen CJ, Yan YJ, Huang CC, Chien JT, Chu CT, Jang JW, Chen TC, Lin SG, Shih RS, Ou-Yang M (2022). Sugariness prediction of *Syzygium samarangense* using convolutional learning of hyperspectral images. Sci. Rep..

[25] Tsai YH, Yan YJ, Li YS, Chang CH, Huang CC, Chen TC, Lin SG, Ou-Yang M (2022). Development and verification of the coaxial heterogeneous hyperspectral imaging system. Rev. Sci. Instrum..

[26] Organisation Internationale de Métrologie Légale. Refractometers for the measurement of the sugar content of fruit juices (1993). https://www.oiml.org/en/files/pdf_r/r108-e93.pdf/@@download/file/R108-e93.pdf. Accessed: July 31, 2023.

[27] Sundararajan, M., Taly, A., and Yan, Q. Axiomatic attribution for deep networks. In *Proceedings of International Conference on Machine Learning*, 3319–3328 (2017).

